# Supplementary material for: Determinants of cigarette/bidi smoking among youth male in rural Mymensingh of Bangladesh: A cross-sectional study
Source: PLoS One. 2020 Dec 28;15(12):e0244335. doi: 10.1371/journal.pone.0244335 (PMC7769457; doi:10.1371/journal.pone.0244335)
Supplement: S1 File — (PDF) [file pone.0244335.s001.pdf]

ত্রিশাল পৌরসভার তরুণ ধূমপায়ীদের ধূমপান সম্পর্কে জ্ঞান, সচেতনতা এবং অভ্যাস ঃ একটি সমীক্ষা

ত্রিশাল পৌরসভার তরুণ ধূমপায়ীদের ধূমপান সম্পর্কে জ্ঞান, সচেতনতা এবং অভ্যাস বিষয়ক একটি গবেষণা পরিচালনার লক্ষ্যে একটি সমীক্ষা। এই প্রশ্নপত্রের মাধ্যমে আপনার নিকট থেকে তথ্য সংগ্রহ করতে আনুমানিক ১৫ মিনিট সময় লাগতে পারে।

দৈবচয়নের ভিত্তিতে নির্বাচিত উত্তরদাতা হিসাবে আপনার দেওয়া তথ্য শুধুমাত্র গবেষণা কাজে ব্যবহার করা হবে এবং প্রদত্ত তথ্যের সার্বিক গোপনীয়তা রক্ষা করা হবে। আপনি আমাদেরকে সাক্ষাৎকার দেওয়া বা না দেওয়ার পূর্ণ স্বাধীনতা রাখেন। আপনি আমাদেরকে সাক্ষাৎকার দিতে রাজি থাকলে আমরা আপনাকে নিচের প্রশ্নগুলো করবো।

উত্তরদাতার আর্থ-সামাজিক অবস্থা সম্পর্কিত তথ্য

১| উত্তরদাতার বয়স (বৎসর)ঃ

২। উত্তরদাতার লিঙ্গঃ ১) পুরুষ ২) মহিলা

৩। উত্তরদাতার শিক্ষাগত যোগ্যতা (পূর্ণ বৎসর) ঃ

৪। উত্তরদাতার পিতার শিক্ষাগত যোগ্যতা (পূর্ণ বৎসর) ঃ

৫। উত্তরদাতার মাতার শিক্ষাগত যোগ্যতা (পূর্ণ বৎসর) ঃ

৬। উত্তরদাতার পেশা ঃ ১) দিনমজুর ২) কৃষিজীবী ৩) চাকুরীজীবি ৪) ব্যবসায়ী ৫) ছাত্র/ছাত্রী ৬) বেকার ৭) অন্যান্য (নির্দিষ্ট করে লিখুন) ঃ

৭। উত্তরদাতার পিতার পেশা ঃ ১) দিনমজুর ২) কৃষিজীবী ৩) চাকুরীজীবি ৪) ব্যবসায়ী ৫) অন্যান্য (নির্দিষ্ট করে লিখুন) ঃ

৮। উত্তরদাতার মাতার পেশা ঃ ১) দিনমজুর ২) কৃষিজীবী ৩) চাকুরীজীবি ৪) ব্যবসায়ী ৫) গৃহিণী ৬) অন্যান্য (নির্দিষ্ট করে লিখুন) ঃ

৯। ছোটবেলায় আপনার পরিবারের অর্থনৈতিক অবস্থা সম্পর্কে আপনি কি মনে করতে পারেন? ১) হ্যাঁ ০) না

১০। হ্যাঁ হলে পরিবারের অর্থনৈতিক অবস্থা কেমন ছিলঃ ১) ধনী ২) মোটামুটি সচ্ছল ৩) গরীব

১১। উত্তরদাতার মাসিক আয় ঃ

১২। পরিবারের মাসিক আয় ঃ

১৩। পরিবারের আয়ের উপর আপনি কি সম্পূর্ণভাবে নির্ভর করেন ? ১) হ্যাঁ ০) না

১৪। পরিবারের সদস্য সংখ্যা ঃ

১৫। গত ৬ মাস যাবত আপনি কি ধূমপান করেন? ১) হ্যাঁ ০) না [উত্তর না হলে ২৩- ২৮ নং স্কিপ করতে হবে]

উত্তরদাতার ধূমপান সম্পর্কে জ্ঞান

১৬। ধূমপান স্বাস্থ্যের জন্য ক্ষতিকর ঃ ১) হ্যাঁ ০) না

১৭। ধূমপান দুরারোগ্য রোগের ঝুঁকি আনতে পারে ঃ ১) হ্যাঁ ০) না

১৮। ধূমপান আশেপাশের অন্য অধূমপায়ীদের জন্য কি ক্ষতিকর ঃ ১) হ্যাঁ ০) না

১৯। আপনি প্রথম কোথায় থেকে ধূমপান সম্পর্কে জেনেছেন? ১) পরিবার ২) বন্ধু/বান্ধব ৩) বিজ্ঞাপন ৪) প্রতিবেশী/আত্মীয় ৫) অন্যান্য (নির্দিষ্ট করে লিখুন) ঃ

২০। আপনি কোথায় ধূমপানের ক্ষতিকর দিক সম্পর্কে জেনেছেন? ১) পরিবার ২) বন্ধু/বান্ধব ৩) বিজ্ঞাপন ৪) প্রতিবেশী/আত্মীয় ৫) অন্যান্য (নির্দিষ্ট করে লিখুন) ঃ

২১। পাবলিক প্লেস এ ধূমপান করা আইনত দণ্ডনীয় অপরাধ এটা জানেন কি? ১) হ্যাঁ ০) না

২২। তামাকজাত পণ্য বিক্রয়ের ক্ষেত্রে বয়সের সীমাবদ্ধতা জানা আছে কি? ১) হ্যাঁ ০) না

২২.১। যদি হ্যাঁ হয় তাহলে কত বছরের নীচের মানুষের কাছে বিক্রি করা যাবেনা?

উত্তরদাতার ধূমপানের অভ্যাস

২৩। আপনি ধূমপান করার প্রেরণা প্রথমে কার নিকট হতে পেয়েছেন? ১) বাবা ২) মা ৩) বন্ধু/বান্ধব ৪) প্রতিবেশী/আত্মীয় ৫) অন্যান্য (নির্দিষ্ট করে লিখুন) ঃ

২৪। ধূমপান করার কারনঃ ১) ধূমপান চাপ এবং দুঃশ্চিন্তা কমায় ২) ধূমপান একটা ফ্যাশান ৩) ধূমপান ভাল অভ্যাস ৪) কৌতূহল বশত ৫) অন্যান্য (নির্দিষ্ট করে লিখুন) ঃ

২৫। প্রথম ধূমপানের সময় আপনার বয়সঃ

২৫.১। আপনার ধূমপান করার ধরণঃ ১) দৈনিক ২) সাপ্তাহিক ৩) মাসিক ৪) অন্যান্য (নির্দিষ্ট করে লিখুন) ঃ

২৬। কত বছর ধরে ধূমপান করেন?

২৭। দিনে কতটি সিগারেট খান?

২৮। দিনে কত টাকা ধূমপানের জন্য খরচ করেন?

২৯। আপনার পিতা-মাতার কেও গত ৬ মাস যাবত ধূমপান করেন কি? ১) কেবল পিতা ২) কেবল মাতা ৩) দুজনেই ৪) কেও না

৩০। পিতা/মাতা ছাড়া গত ৬ মাস যাবত পরিবারের অন্য কেও ধূমপান করে? ১) ভাই ২) দাদা

৩) নানা ৪) অন্যান্য (নির্দিষ্ট করে লিখুন) ঃ

৩১। আপনার ঘনিষ্ট বন্ধুদের কেও গত ৬ মাস যাবত ধূমপান করেন কি? ১) কেও কেও ২) প্রায় সবাই ৩) কেও না

ধূমপান সম্পর্কে জ্ঞান, সচেতনতা

৩২। সিগারেটের বিজ্ঞাপন বন্ধ ধূমপান কমাতে পারে? ১) হ্যাঁ ০) না

৩৩। পাবলিক প্লেস ধূমপানমুক্ত হওয়া উচিতঃ ১) হ্যাঁ ০) না

৩৪। সিগারেট এবং তামাকজাত পণ্যের কর বৃদ্ধি করা উচিত ঃ ১) হ্যাঁ ০) না

৩৫। ধূমপানের ক্ষতিকর দিক নিয়ে শিক্ষা প্রদান করা উচিতঃ ১) হ্যাঁ ০) না

৩৬। ধূমপান ত্যাগ করা স্বাস্থ্যের জন্য ভালঃ ১) হ্যাঁ ০) না

৩৭। আপনি কখনও ধূমপান ছেড়ে দেবার চেষ্টা করেছেন? ১) হ্যাঁ ০) না

৩৮। যদি হ্যাঁ হয় তবে ছাড়তে পারেননি কেন? ১) ছাড়া খুব কঠিন ২) ইচ্ছা শক্তির অভাব ৩) অন্যান্য (নির্দিষ্ট করে লিখুন) ঃ

তথ্য প্রদানের জন্য আপনাকে অনেক ধন্যবাদ।
