# Supplementary material for: Determinants of cigarette/bidi smoking among youth male in rural Mymensingh of Bangladesh: A cross-sectional study
Source: PLoS One. 2020 Dec 28;15(12):e0244335. doi: 10.1371/journal.pone.0244335 (PMC7769457; doi:10.1371/journal.pone.0244335)
Supplement: S2 File — (PDF) [file pone.0244335.s002.pdf]

Knowledge, Awareness and Practices among Youth Smokers in Trishal Upazila under Mymensingh District: A Micro-Survey Study

This is a survey on knowledge, awareness and practices of smoking among youth in Trishal Upazila. Your information will be gathered through this questionnaire, and it will take approximately 15 minutes.

You are selected on the basis of simple random sampling, your information will be kept strictly confidential, and the data will be utilized only for research purpose. You have full freedom to either provide or withdraw from this interview. If you provide your verbal consent, we will ask the following questions.

Socio-Economic Characteristics of the Respondents

1. Age of the respondent (in years):
2. Sex of the respondent: 1) Male 2) Female
3. Education stats of the respondent (in years):
4. Father's education (in years):
5. Mother's education (in years):
6. Occupation status of the respondent: 1) Day labourer 2) Farmer 3) Service holder 4) Business 5) Student 6) Unemployed 7) Others (mention):
7. Occupation status of father: 1) Day labourer 2) Farmer 3) Service holder 4) Business 5) Others (mention):
8. Occupation status of mother: 1) Day labourer 2) Farmer 3) Service holder 4) Business 5) Housewife 6) Others (mention):
9. Can you remember the financial status of your family at your childhood? 1) Yes 0) No
10. If yes, what was your family's financial status during your childhood:1) rich 2) about average 3) poor?
11. Respondent's monthly income:
12. Family's monthly income:
13. Are you totally dependent on your family's income? 1) Yes 0) No
14. Number of family members:
15. Have you been smoking for last six months? 1) Yes 0) No [Skip the question no. 23-28, if the answer is No]

Knowledge of Smoking

16. Smoking is harmful to health: 1) Yes 0) No
17. Smoking brings the risk of chronic disease: 1) Yes 0) No
18. Is smoking harmful to other nearby non-smokers: 1) Yes 0) No
19. Sources of information about smoking: 1) Family 2) Friends 3) Advertisement from different media 4) Neighbour/relatives 5) Others (mention)

20. Sources of information about harmful effects of smoking: 1) Family 2) Friends 3) Advertisement from different media 34) Neighbour/relatives 4) Advertisement from different media 5) Others (mention)
21. Do you know smoking in a public place is an offense? 1) Yes 0) No
22. Do you know the restriction on age limit to sell tobacco products: 1) Yes 0) No

22.1 If yes, then what is the age limit?

Practice of Smoking

23. Who do you inspire smoking for the first time? 1) Father 2) Mother 3) Friends 4) Neighbours/relatives 5) Others (mention)
24. Reasons for smoking: 1) Smoking reduces stress and anxiety 2) Smoking is fashionable 3) Smoking is good habit 4) Curious to try 5) Others (mention)
25. Age at first smoke:

25.1 Type of your smoking: 1) Daily 2) Weekly 3) Monthly 4) Others (mention)

26. How long have you been smoking?
27. How many cigarette do you smoke per day?
28. Daily expenditure per day on smoking:
29. Have your parents been smoking for last six months? 1) Only father 2) Only mother 3) Both 4) None
30. Has anyone in the family been smoking for last six months, except for parents? 1) Brother 2) Paternal grandfather 3) Maternal grandfather 4) Others (mention)
31. Have your closest friends been smoking for last six months? 1) Some of them 2) Almost all of them 3) None of them

Awareness of Smoking

32. Can banning on cigarette advertisement reduce smoking? 1) Yes 0) No
33. Public places should be smoke free: 1) Yes 0) No
34. Should taxes on cigarette and other tobacco products be increased? 1) Yes 0) No
35. Should educational lessons on the detrimental effects of smoking be provided? 1) Yes 0) No
36. Quitting smoking is good for health: 1) Yes 0) No
37. Do you make any attempt to quit smoking? 1) Yes 0) No
38. If yes, reason for not being able to quit? 1) Difficult to quit 2) Lack of willpower 3) Others (mention):

Thank you very much for proving information.
