# Supplementary material for: Determinants of cigarette/bidi smoking among youth male in rural Mymensingh of Bangladesh: A cross-sectional study
Source: PLoS One. 2020 Dec 28;15(12):e0244335. doi: 10.1371/journal.pone.0244335 (PMC7769457; doi:10.1371/journal.pone.0244335)
Supplement: Attachment — Submitted filename: PONE-D-20-25908_reviewer Determinants of smoking among youth male in Bangladesh.pdf [file pone.0244335.s004.pdf]

PLOS ONE

Determinants of cigarette/ bidi smoking among youth male in rural Mymensingh of Bangladesh: A cross-sectional study

--Manuscript Draft--

**Manuscript Number:**	PONE-D-20-25908
**Article Type:**	Research Article
**Full Title:**	Determinants of cigarette/ bidi smoking among youth male in rural Mymensingh of Bangladesh: A cross-sectional study
**Short Title:**	Determinants of smoking among youth male in Bangladesh
**Corresponding Author:**	Md. Ismail Tareque, Ph.D.<br>University of Rajshahi<br>Rajshahi, BANGLADESH
**Keywords:**	Smoking; youth; rural; determinants; Bangladesh
**Abstract:**	Background<br><br>Smoking cigarette/ bidi, is a serious health threat, causes preventable premature morbidity and mortality. Higher prevalence of smoking among the youth hampers a country's development, as the youth are the main drives of socio-economic development. An effective understanding of factors associated with youth smoking is precious to prevent youth smoking. This study aims to identify the determinants of smoking cigarette/bidi among the youth male of the rural areas of Mymensingh district in Bangladesh.<br><br>Methods<br><br>Data came from a secondary source. A total of 385 youth male aged 15-24 years were interviewed face-to-face from the rural areas of Mymensingh district in Bangladesh. Univariate distribution, chi-square tests, and binary logistic regression model were employed to identify the factors associated with smoking cigarette/bidi among the youth male.<br><br>Results<br><br>The prevalence of smoking cigarette/bidi among the youth male is 40.3%. Age, occupation, monthly income, family's monthly income, cigarette/ bidi smoking status of father, brother and close friends, and knowledge about harmfulness of smoking are revealed as the determinants of cigarette/ bidi smoking. For instance, the odds of being smoker for those aged 18-20 years is 1.78 times [95% CI: 0.77-4.11], and for those aged 21-24 years is 5.54 times [2.25-13.61] than those aged 15-17 years. Business owner is less likely (Odds Ratio [OR]: 0.15 [0.03-0.69]) to smoke than the day labourer. Having smoker fathers (OR: 2.24 [1.25-3.98]), smoker brothers (OR: 3.03 [1.46-6.29]), smoker friends (OR: 10.65 [5.89-19.25]) are significantly associated with smoking cigarette/ bidi.<br><br>Conclusion<br><br>As the first study, it provides the determinants of cigarette/ bidi smoking among youth male of the rural areas of Mymensingh district in Bangladesh. Relevant authorities are suggested to consider the study's findings and recommendations to revise the existing smoking policies so that smoking among youth can be prevented for future development of the country.
**Order of Authors:**	K. M. Mustafizur Rahman
	Md. Ismail Tareque, Ph.D.
**Additional Information:**	
**Question**	**Response**

Powered by Editorial Manager® and ProduXion Manager® from Aries Systems Corporation

**Financial Disclosure**<br><br>Enter a financial disclosure statement that describes the sources of funding for the work included in this submission. Review the submission guidelines for detailed requirements. View published research articles from *PLOS ONE* for specific examples.<br><br>This statement is required for submission and **will appear in the published article** if the submission is accepted. Please make sure it is accurate.<br><br>**Unfunded studies**<br>Enter: *The author(s) received no specific funding for this work.*<br><br>**Funded studies**<br>Enter a statement with the following details:<br>• Initials of the authors who received each award<br>• Grant numbers awarded to each author<br>• The full name of each funder<br>• URL of each funder website<br>• Did the sponsors or funders play any role in the study design, data collection and analysis, decision to publish, or preparation of the manuscript?<br>• **NO** - Include this sentence at the end of your statement: *The funders had no role in study design, data collection and analysis, decision to publish, or preparation of the manuscript.*<br>• **YES** - Specify the role(s) played.<br><br>* typeset	The author(s) received no specific funding for this work.
**Competing Interests**<br><br>Use the instructions below to enter a competing interest statement for this submission. On behalf of all authors, disclose any competing interests that could be perceived to bias this work—acknowledging all financial support and any other relevant financial or non-financial competing interests.<br><br>This statement **will appear in the published article** if the submission is accepted. Please make sure it is	The authors have declared that no competing interests exist.

Powered by Editorial Manager® and ProduXion Manager® from Aries Systems Corporation

accurate. View published research articles from *PLOS ONE* for specific examples.<br><br>**NO authors have competing interests**<br><br>Enter: *The authors have declared that no competing interests exist.*<br><br>**Authors with competing interests**<br><br>Enter competing interest details beginning with this statement:<br><br>*I have read the journal's policy and the authors of this manuscript have the following competing interests: [insert competing interests here]*<br><br>* typeset	
**Ethics Statement**<br><br>Enter an ethics statement for this submission. This statement is required if the study involved:<br><br>• Human participants<br>• Human specimens or tissue<br>• Vertebrate animals or cephalopods<br>• Vertebrate embryos or tissues<br>• Field research<br><br>Write "N/A" if the submission does not require an ethics statement.<br><br>General guidance is provided below. Consult the submission guidelines for detailed instructions. **Make sure that all information entered here is included in the Methods section of the manuscript.**	The study design and funding for the project titled ‘‘Knowledge, awareness and practices among youth smokers in Trishal Upazila under Mymensingh district: A micro-survey study’’ were approved by the Research and Extension Center, Jatiya Kabi Kazi Nazrul Islam University, Bangladesh. All respondents were asked to provide verbal consent after being read a document emphasizing voluntary participation in the project. Any identifying information was removed. As the de-identified data for this study came from secondary source, this study does not require ethical approval.

**Format for specific study types**<br><br>**Human Subject Research (involving human participants and/or tissue)**<br>• Give the name of the institutional review board or ethics committee that approved the study<br>• Include the approval number and/or a statement indicating approval of this research<br>• Indicate the form of consent obtained (written/oral) or the reason that consent was not obtained (e.g. the data were analyzed anonymously)<br><br>**Animal Research (involving vertebrate animals, embryos or tissues)**<br>• Provide the name of the Institutional Animal Care and Use Committee (IACUC) or other relevant ethics board that reviewed the study protocol, and indicate whether they approved this research or granted a formal waiver of ethical approval<br>• Include an approval number if one was obtained<br>• If the study involved *non-human primates*, add *additional details* about animal welfare and steps taken to ameliorate suffering<br>• If anesthesia, euthanasia, or any kind of animal sacrifice is part of the study, include briefly which substances and/or methods were applied<br><br>**Field Research**<br><br>Include the following details if this study involves the collection of plant, animal, or other materials from a natural setting:<br>• Field permit number<br>• Name of the institution or relevant body that granted permission	
**Data Availability**<br><br>Authors are required to make all data underlying the findings described fully available, without restriction, and from the time of publication. PLOS allows rare exceptions to address legal and ethical concerns. See the PLOS Data Policy and FAQ for detailed information.	Yes - all data are fully available without restriction

A Data Availability Statement describing where the data can be found is required at submission. Your answers to this question constitute the Data Availability Statement and **will be published in the article**, if accepted.<br><br>**Important:** Stating 'data available on request from the author' is not sufficient. If your data are only available upon request, select 'No' for the first question and explain your exceptional situation in the text box.<br><br>Do the authors confirm that all data underlying the findings described in their manuscript are fully available without restriction?	
**Describe where the data may be found in full sentences. If you are copying our sample text, replace any instances of XXX with the appropriate details.**<br><br>• If the data are **held or will be held in a public repository**, include URLs, accession numbers or DOIs. If this information will only be available after acceptance, indicate this by ticking the box below. For example: *All XXX files are available from the XXX database (accession number(s) XXX, XXX.*).<br>• If the data are all contained **within the manuscript and/or Supporting Information files**, enter the following: *All relevant data are within the manuscript and its Supporting Information files.*<br>• If neither of these applies but you are able to provide **details of access elsewhere**, with or without limitations, please do so. For example:<br><br>*Data cannot be shared publicly because of [XXX]. Data are available from the XXX Institutional Data Access / Ethics Committee (contact via XXX) for researchers who meet the criteria for access to confidential data.*<br><br>*The data underlying the results presented in the study are available from (include the name of the third party*	The data will be made available only after acceptance of the manuscript. The data will be shared through email.

and contact information or URL).<br>• This text is appropriate if the data are owned by a third party and authors do not have permission to share the data.<br><br>* typeset	
Additional data availability information:	

Manuscript
Click here to access/download;Manuscript;clean_manuscript_1st_submissio

Determinants of cigarette/ bidi smoking among youth male in rural Mymensingh of Bangladesh: A cross-sectional study

K. M. Mustafizur Rahman [1], Md. Ismail Tareque [2*]

[1] Department of Population Science, Jatiya Kabi Kazi Nazrul Islam University, Trishal-2220, Mymensingh, Bangladesh

[2] Department of Population Science and Human Resource Development, University of Rajshahi, Rajshahi 6205, Bangladesh

E-mail

K. M. Mustafizur Rahman: nishan_hrd@yahoo.com

Md. Ismail Tareque: tarequemi_pops@ru.ac.bd

ORCID ID

K. M. Mustafizur Rahman: 0000-0002-3817-9048

Md. Ismail Tareque: 0000-0002-6507-1171

* Corresponding author

Email: tarequemi_pops@ru.ac.bd (MIT)

tareque_pshd@yahoo.com (MIT)

// Abstract

Background

Smoking cigarette/ bidi, is a serious health threat, causes preventable premature morbidity and mortality. Higher prevalence of smoking among the youth hampers a country's development, as the youth are the main drives of socio-economic development. An effective understanding of factors associated with youth smoking is precious to prevent youth smoking. This study aims to identify the determinants of smoking cigarette/bidi among the youth male of the rural areas of Mymensingh district in Bangladesh.

Methods

Data came from a secondary source. A total of 385 youth male aged 15-24 years were interviewed face-to-face from the rural areas of Mymensingh district in Bangladesh. Univariate distribution, chi-square tests, and binary logistic regression model were employed to identify the factors associated with smoking cigarette/bidi among the youth male.

Results

The prevalence of smoking cigarette/bidi among the youth male is 40.3%. Age, occupation, monthly income, family's monthly income, cigarette/ bidi smoking status of father, brother and close friends, and knowledge about harmfulness of smoking are revealed as the determinants of cigarette/ bidi smoking. For instance, the odds of being smoker for those aged 18-20 years is 1.78 times [95% CI: 0.77-4.11], and for those aged 21-24 years is 5.54 times [2.25-13.61] than those aged 15-17 years. Business owner is less likely (Odds Ratio [OR]: 0.15 [0.03-0.69]) to smoke than the day labourer. Having smoker fathers (OR: 2.24 [1.25-3.98]), smoker brothers (OR: 3.03 [1.46-6.29]), smoker friends (OR: 10.65 [5.89-19.25]) are significantly associated with smoking cigarette/ bidi.

Conclusion

As the first study, it provides the determinants of cigarette/ bidi smoking among youth male of the rural areas of Mymensingh district in Bangladesh. Relevant authorities are suggested to consider the study’s findings and recommendations to revise the existing smoking policies so that smoking among youth can be prevented for future development of the country.

Key words

Smoking; youth; rural; determinants; Bangladesh.

Introduction

Tobacco use is a significant public health concern. Globally, more than 7 million people die due to direct tobacco use, and around 1.2 million non-smokers die due to being exposed to second-hand smoke [1]. All forms of tobacco are harmful, and there is no safe level of exposure to tobacco [1]. Cigarette/ bidi smoking is the most common form of tobacco use among men in Bangladesh, where 43% of adults use some form of tobacco, 45% of men and only 1.5% of women smoke cigarettes [2]. Despite having ban on the sales of tobacco to and by minors, particularly those aged <18 years, in Bangladesh national tobacco control law, university male students are found to initiate smoking at an average age of 17.8 years [3]. Evidences demonstrate that majority of the people try their first cigarette before the age of 18 and become daily smokers in adolescents. For instance, most of the European start smoking before the age of 18 and often transit to regular smokers during young adulthood [4]. In the United States, about 90% of all regular smokers begin smoking at or before age 18, and hardly any regular smoker tries the first cigarette outside of childhood [5,6]. When people start smoking at an earlier age, the duration of smoking must be longer for the people at adulthood. Due to the prolonged smoking, a wide range of diseases can accelerate the premature morbidity and mortality among the adults [7]. The longer duration of smoking creates more miserable conditions to their lives, families, societies and the country, especially for developing country like Bangladesh; because cigarette smoking harms almost every organ of human body, causes many diseases, and reduces the health of smokers in general [8]. Therefore, it is imperative to study the factors that are associated with smoking, particularly among the youth, in Bangladesh. Identification of modifiable factors will provide the possibilities to prevent smoking among the youth.

Bangladesh has made a considerable progress in socio-economic and demographic indicators over the last few decades [9]. And, the socio-economic and demographic indicators

are progressing in the right path to achieve the desired level of prospective socio-economic development in the country. However, the progress could stay behind the desired level due to the use of tobacco among the youth, as the use of tobacco has high impact on growing economy and high expenditure on health [10]. Bangladesh is passing through its golden opportunity of demographic dividend where youth remains the central to such development. Therefore, smoking among youth is alarming issue to be taken care of for the future development of the country.

According to the Global Adult Tobacco Survey, in Bangladesh, around 22 million people are smoking different types of tobacco products, among them 12% people aged 15-24 years (considered youth in the current study; discussed in the Methods section) are consuming some form of tobacco [11]. The number of tobacco users, particularly among the youth, is increasing day by day due to availability of cheap tobacco products, poor status of tobacco control regulations, and weak enforcement of such regulations [3,12]. Youth is an important age group as healthy youth leads to healthy future. They are the main instruments for future development of the country. Higher incidence of smoking among the youth surely hampers the future development. Hence, it is indispensable to restrict the youth from smoking. In different settings, factors associated with cigarette smoking among the youth includes exposure to smokers (elders, parents, friends, teachers), availability of tobacco, low socio-economic status, low self-esteem, poor academic performances [13,14]. Although there are studies on the determinants of smoking around the world [15–20], there is a paucity of research to identify the factors responsible for smoking among the youth in Bangladesh. In fact, the majority of the existing studies have been limited to the prevalence and predictors of tobacco use among the university students. It is essential to identify the determinants of youth smoking in Bangladesh. Thus, the aim of this study is to identify the determinants of smoking among the youth in Bangladesh. It is believed that findings of the current study will be

helpful for Bangladeshi policy makers in formulating strong measures along with revising or redrawing the existing policies to discourage smoking among the youth in Bangladesh.

Methods

Data

We utilized secondary data for this study. The secondary data came from the project titled ‘‘Knowledge, awareness and practices among youth smokers in Trishal Upazila under Mymensingh district: A micro-survey study’’, funded by the Research and Extension Center, Jatiya Kabi Kazi Nazrul Islam University, Bangladesh. In brief, the data for the project were collected from youth male aged 15-24 years (according to United Nations, individuals aged 15-24 years are youth [21]) from the rural areas of Trishal Upazila of Mymensingh district of Bangladesh from November 25, 2019 to December 15, 2019. The upazila is about 20 kilometers away from the divisional town of Mymensingh, and about 90 kilometers away from the capital city of Dhaka. The Upazila consists of 1 municipality and 12 unions. Using simple random sampling technique, at first one union (namely, Trishal Union) from Trishal Upazila, and then five wards (Ward 1, 2, 3, 8 and 9) from Trishal Union were selected. Finally, using systematic random sampling technique, data from 385 youth males were collected from the selected wards. Based on the total number of youth male in the selected wards, obtained from the Bangladesh Population and Housing Census 2011 [22], the data were collected, and during data collection one of every fifth male youth was interviewed in the selected wards. Consequently, 53 youth male from Ward 1, 81 from Ward 2, 74 from Ward 3, 91 from Ward 8, and 86 from Ward 9 were interviewed. A well-structured questionnaire with demographic and socio-economic questions was administered face-to-face to 385 youth males. From socio-cultural and religious points of view, smoking among female

are still beyond the consideration in Bangladesh. Therefore, only males were considered for the project.

Outcome variable

Current smoking status of the respondents (youth males aged 15-24 years) is considered as the outcome variable, which was dichotomized by assigning 1 if a respondent had been cigarette/ bidi smoking for last six months prior to the survey, and 0 for otherwise.

Explanatory variables

A wide range of individual and household characteristics, reported to be associated with smoking status among the youth in previous studies [20,23–27], were included in the current study. Individual's characteristics include respondent's current age (classified as 15-17, 18-20, or 21-24 years), education (categorized 0 to 5 years of schooling as below secondary, 6 to 10 years of schooling as secondary, or 11+ years of schooling as higher secondary and above), occupation (categorized as day labourer, service, business, students, or unemployed), monthly income (no income, <5000 Bangladesh currency Taka [BDT], or ≥5000 BDT), financial dependency on the family (classified as dependent or independent), knowledge about the risks of disease– whether smoking brings the risk of chronic disease (no or yes), and knowledge about the harmfulness– whether smoking is harmful to other non-smokers (no or yes). Household's characteristics include education of father (categorized as below secondary, secondary, or higher secondary and above), education of mother (categorized as below secondary, secondary, or higher secondary and above), family income (<15000 BDT, 15000-25000 BDT, or >25000 BDT), current smoking status of father (no or yes), current smoking status of brothers (no or yes). Additionally, current smoking status of close friends (no or yes) was considered as explanatory variable, as youths are significantly influenced by their close friends [28]. The respondent's father, brother and close friends were

considered as smokers if they had been cigarette/ bidi smoking for last six months prior to the survey.

Statistical analysis

After descriptive statistics of the study sample, chi-square tests were used to identify differences in the percentage of smokers by the explanatory variables (detailed above). All variables significant in chi-square tests at level $p<0.20$ were included in regression analysis. Finally, a binary logistic regression model was used to identify the factors that are influential in determining the smoking status of the respondents. The statistical significance of all analysis was set at $p<0.05$.

Ethical considerations

The study design and funding for the project titled ‘‘Knowledge, awareness and practices among youth smokers in Trishal Upazila under Mymensingh district: A micro-survey study’’ were approved by the Research and Extension Center, Jatiya Kabi Kazi Nazrul Islam University, Bangladesh. All respondents were asked to provide verbal consent after being read a document emphasizing voluntary participation in the project. Any identifying information was removed. As the de-identified data for this study came from secondary source, this study does not require ethical approval.

Results

Table 1 presents the basic characteristics of the study participants. The average age is 18.9 years. Among the participants, 40% are 15-17 years, 28.8% are 18-20 years, and 31.2% are 21-24 years old. Average year of schooling is 9.6, and only 12.5% of respondents are with below secondary level of education. Respondents' fathers are with more years of education than their mothers (average year of education: father 7.3 years and mother 5.9 years). Majority of the respondents are students (67%). Although majority (66.8%) has no income,

the average monthly income is BDT 4,439. The average monthly income of respondent's family is BDT 22634, with around half (46%) of family's income in between BDT 15000-25000. 71.7% of respondents depend on their family for their financial supports while remaining 28.3% do not depend on their family for their financial supports. 40.3% of respondents are currently cigarette/ bidi smoking, 57.4% of the respondents' fathers are currently cigarette/ bidi smoking, 17.7% of respondents' brothers are currently cigarette/ bidi smoking, and 36.1% of the respondents' close friends are currently cigarette/ bidi smoking. Overwhelming majority of the respondents believe that smoking brings the risks of chronic diseases, and smoking is harmful to other non-smokers.

<Table 1 about here>

Table 2 [illegible]alyzes the bivariate association between cigarette/ bidi smoking status and socio-demographics, and shows the variation in percentage of smokers by socio-demographics. A significant association between cigarette/ bidi smokers and age group was found; percentage of cigarette/ bidi smokers increase with the increase in age ($p<0.001$). Respondents who are with below secondary education, day labourers, with monthly income BDT<5,000, with monthly family income BDT>25,000, financially independent from their family are more likely to smoke cigarette/ bidi than their counterparts. Respondents whose fathers, brothers and close friends are cigarette/ bidi smokers are also more likely to smoke cigarette/ bidi than the respondents whose fathers, brothers and close friends are non-smokers. Respondents who believe that smoking brings the risks of chronic diseases and is harmful to other non-smokers are less likely to smoke cigarette/ bidi than their counterparts.

<Table 2 about here>

Table 3 shows the results of binary logistic regression model that yields the odds of being cigarette/ bidi smokers by categories of the explanatory variables. The likelihood of being cigarette/ bidi smokers increases with the increase in age. The odds of being cigarette/

bidi smokers for those in age group 18-20 years is 1.78 times (95% confidence interval: 0.77-4.11) and for those in age group 21-24 years is 5.54 times (2.25-13.61) than those in age group 15-17 years. The odds of being cigarette/ bidi smokers for business owner is 0.15 times (0.03-0.69) than day labourer. Compared to respondents with no income, respondents with monthly income (either <BDT 5,000 or ≥BDT 5,000) are more likely to smoke cigarette/ bidi. Respondents coming from higher economic status (i.e. family's monthly income ≥BDT 15,000) have higher odds of being cigarette/ bidi smokers than those coming from lower economic status (i.e. family's monthly income <BDT 15,000). Respondents whose fathers are cigarette/ bidi smokers are more likely to smoke cigarette/ bidi than those having non-smoker fathers; also whose brothers and close friends are cigarette/ bidi smokers are more likely to smoke cigarette/ bidi than their counterparts. Respondents who believe that smoking is harmful to other non-smokers are significantly less likely to smoke cigarette/ bidi than their counterparts.

<Table 3 about here>

Discussion

To the best of our knowledge, this is the first study that reveals the determinants of cigarette/ bidi smoking among youth male in Bangladesh. The small-scale cross-sectional data exhibit that four in 10 youth male are cigarette/ bidi smokers in Trishal Upazila of Mymensingh district. The determinants of cigarette/ bidi smoking among youth male include age, occupation, monthly income, family's monthly income, cigarette/ bidi smoking status of father, brother and close friends, and knowledge about harmfulness of smoking.

Higher age, particularly 21-24 years are associated with higher likelihood of cigarette/ bidi smoking among youth male in the current study. Age is also found as one of the determinants of smoking among youth in Ethiopia [18,29]. This might be due to the fact that the possibility of being involved in various risky practices, particularly among youth male,

increases with the increase in age. Also, in Bangladesh, older youth male have some cash in their hands with which they are capable of buying cigarette/ bidi. It is particularly evident in the study area, where 69% of the respondents have some income.

Day labourers have the highest odds of smoking cigarette/ bidi in the current study. In the United States, blue-collar workers, specifically construction workers in the absence of their workplace rules against smoking, are more likely to be smokers than white-collar workers [30]. And, workplace smoking restrictions, mainly smoke-free policies are reported promising for reducing smokers in Canada [31]. In Bangladesh, while service holders, businessmen and students need to comply with smoking restrictions at workplaces/ schools, day labourers face almost no smoking restriction at workplaces, and unemployed youth have their limitations in expending money on smoking. No smoking restriction at workplaces could be the possible reason for the highest likelihood of being smokers among day labourers in the study area. Restricting smoking for day labourers at workplaces may reduce prospective smokers among youth male in Bangladesh.

There is an independent positive association of personal income with smoking among adolescents aged 14-17 years in six European countries [32]. Economic inequality has also direct correspondence with tobacco use [33]. Youth male with personal income, compared to those with no income, have higher likelihood of cigarette/ bidi smoking in the current study, which is in a line with the previous studies [34,35]. When youth have extra pocket money, they spend the money for their recreation. And, youth male, particularly with their friends, consider smoking as a pastime and therefore expend on smoking cigarette/ bidi in Bangladesh. Providing more valuable options, e.g. recreational football game to the youth would not only reduce number of smokers but also keep the youth fit and active in Bangladesh.

Consistent with previous literature [36], respondents coming from higher economic status have higher likelihood of smoking cigarette/ bidi in our study areas. While households with higher income are able to spend more money on their children, households with lower income struggle to manage their family expenditure. The youth from higher family income receive more pocket money than their counterparts, and may expend on smoking cigarette/ bidi.

Paternal smoking, specifically father's smoking was reported to increase the risk of youth smoking [37]. The current study also finds higher likelihood of smoking cigarette/ bidi among youth male whose fathers are smokers. In a similar vein, brother's smoking is found to increase the risk of youth smoking in the study areas. Smoking among male is found to be associated with the smoking behaviour of siblings in different settings [38,39]. These findings indicate that smoking behaviour passes from one generation to another as well as within the same generation. The possible reason might be that the children who grow up by seeing their fathers and brothers smoking, those children may try to make a trail at some later points in time, and may become a regular smoker at their youth stages. Therefore, to prevent smoking among youth male in a family, the older adults– primarily fathers and male siblings of the family should stop smoking in Bangladesh.

Friend smoking is by far the strongest factor to increase the risk of youth smoking in different settings [18,29,37,38,40,41]. The current study also exhibits higher likelihood of smoking cigarette/ bidi among the respondents whose friends are smokers, compared those with non-smoker friends. Since this study, along with the extant literature, yields the greater peer influences on youth smoking, we suggest families to pay a great attention to their youth members to prevent them from smoking. Similar to the findings of the studies [42,43], the respondents who believe that smoking is harmful to other non-smokers are less likely to smoke cigarette/ bidi in the study areas. Awareness needs to be created through different

channels about deadly hazardous effects of smoking and its harmful consequences not only for the smokers and their families but also for the nation as a whole. In this regard, educational institutions and mass media may play an important role.

The current study has some limitations. Being a cross-sectional one, this study does not permit casual association of the explanatory variables with the outcome variable. The data come from rural areas of Trishal Upazila of Mymensingh district, and thus may not be generalizable to entire youth male in Bangladesh. Despite the limitations, as the pioneer study, it demonstrates a detailed analysis of the various socio-demographic determinants of youth smoking, which is generalizable to rural youth male of Mymensingh district in Bangladesh.

Conclusion

In sum, as the first study, it provides the determinants of cigarette/ bidi smoking among youth male of the rural areas of Mymensingh district. Based on the above discussion, carefully planning should be made to prevent smoking among youth, as the youth are the main instruments for future development of the country. Relevant authorities may consider the following five recommendations in formulating strong enforcement measures along with revising or redrawing the existing smoking policies to discourage smoking among the youth. The recommendations are: (i) restricting smoking for day labourers at workplaces, (ii) providing more valuable options to the youth for pastimes, e.g. recreational football game, (iii) families to pay due attention to their youth members to prevent them from smoking, (iv) the older adults of the families– primarily fathers and male siblings stop smoking, and (v) educational institutions and mass media create awareness about the hazardous effects and harmful consequences of smoking not only for the smokers and their families but also for the entire country.

Acknowledgements

The authors gratefully acknowledge the Research and Extension Center, Jatiya Kabi Kazi Nazrul Islam University, Bangladesh, for providing the scope to conduct this study.

Funding

The authors received no specific funding for this work.

Conflict of interest

The author(s) declare no potential conflicts of interest with respect to the research, authorship, and/ or publication of this article.

References

1. World Health Organization. Tobacco. 2020 [cited 10 Aug 2020]. Available: https://www.who.int/news-room/fact-sheets/detail/tobacco
2. World Health Organization. WHO | Bangladesh – National tobacco control law amended. In: WHO [Internet]. World Health Organization; 2013 [cited 7 Aug 2020]. Available: http://www.who.int/fctc/implementation/news/news_ban/en/
3. Hossain S, Hossain S, Ahmed F, Islam R, Sikder T, Rahman A. Prevalence of tobacco smoking and factors associated with the initiation of smoking among university students in Dhaka, Bangladesh. Central Asian journal of global health. 2017;6.
4. Eurobarometer S. Attitudes of Europeans towards tobacco and electronic cigarettes. TNS Opinion Social. 2015;429.
5. Giovino GA. Epidemiology of tobacco use in the United States. Oncogene. 2002;21: 7326–7340.

6. Johnston LD, Miech RA, O'Malley PM, Bachman JG, Schulenberg JE, Patrick ME. Monitoring the Future National Survey Results on Drug Use, 1975-2018: Overview, Key Findings on Adolescent Drug Use. Institute for Social Research. 2019.
7. Al-Kubaisy W, Abdullah NN, Al-Nuaimy H, Kahn SM, Halawany G, Kurdy S. Factors associated with smoking behaviour among university students in Syria. Procedia-Social and Behavioral Sciences. 2012;38: 59–65.
8. CDC Tobacco Free. Health Effects of Cigarette Smoking. In: Centers for Disease Control and Prevention [Internet]. 2019 [cited 10 Aug 2020]. Available: https://www.cdc.gov/tobacco/data_statistics/fact_sheets/health_effects/effects_cig_smoking/index.htm
9. Ullah SMA, Rahman KMM, Islam MT. Development of Selected Sectors under Democratic Governments in Bangladesh: A Comparative Analysis. KILA Journal of Local Governance. 2018;5: 21–28.
10. World Health Organization. The World Health Report 1999-making a difference. Chapter 5-Combating the tobacco epidimic. World Health Organization; 1999 pp. 65–69. Available: https://www.who.int/whr/1999/en/whr99_ch5_en.pdf?ua=1
11. World Health Organization. Global adult tobacco survey: Bangladesh Report 2009. 2009.
12. Kamimura A, Ahmmad Z, Pye M, Gull B. Peer smoking and smoking-related beliefs among college students in Bangladesh. Journal of Preventive Medicine and Public Health. 2018;51: 51.
13. Bandason T, Rusakaniko S. Prevalence and associated factors of smoking among secondary school students in Harare Zimbabwe. Tobacco Induced Diseases. 2010;8: 12.
14. Escario JJ, Wilkinson AV. Visibility of smoking among schoolteachers in Spain and associations with student smoking: a cross-sectional study. BMJ open. 2018;8.

15. Doku D, Koivusilta L, Raisamo S, Rimpelä A. Do socioeconomic differences in tobacco use exist also in developing countries? A study of Ghanaian adolescents. BMC Public Health. 2010;10: 758.
16. Nguyen CV. Demographic and socio-economic determinants of smoking behavior: evidence from Vietnam. Economics Bulletin. 2012;32: 2300–2312.
17. Nketiah-Amponsah E, Afful-Mensah G, Ampaw S. Determinants of cigarette smoking and smoking intensity among adult males in Ghana. BMC public health. 2018;18: 941.
18. Reda AA, Moges A, Yazew B, Biadgilign S. Determinants of cigarette smoking among school adolescents in eastern Ethiopia: a cross-sectional study. Harm reduction journal. 2012;9: 39.
19. Smet B, Maes L, De Clercq L, Haryanti K, Winarno RD. Determinants of smoking behaviour among adolescents in Semarang, Indonesia. Tobacco control. 1999;8: 186–191.
20. Van Loon AJM, Tijhuis M, Surtees PG, Ormel J. Determinants of smoking status: cross-sectional data on smoking initiation and cessation. The European Journal of Public Health. 2005;15: 256–261.
21. United Nations. Secretary General's Report to the General Assembly,(A/36/215). United Nations New York, NY; 1981.
22. Bangladesh Bureau of Statistics. Bangladesh Population and Housing Census 2011; Zila Report: Mymensingh. Statistics and Informatics Division (SID), Ministry of Planning, Governments of the People's Republic of Bangladesh; 2015. Available: http://203.112.218.65:8008/WebTestApplication/userfiles/Image/PopCenZilz2011/Zila_Mymensingh.pdf

23. Chhakchhuak L, Hriatpuii L, Chhakchhuak L. Socio-economic status as predictors of initiation and persistence of youth smoking in Mizoram. Tobacco Induced Diseases. 2018;16: pp. 367–367.
24. Fernandez E, Garcia M, Schiaffino A, Borras JM, Nebot M, Segura A. Smoking initiation and cessation by gender and educational level in Catalonia, Spain. Preventive medicine. 2001;32: 218–223.
25. Linetzky B, Mejia R, Ferrante D, De Maio FG, Diez Roux AV. Socioeconomic status and tobacco consumption among adolescents: a multilevel analysis of Argentina's Global Youth Tobacco Survey. Nicotine & Tobacco Research. 2012;14: 1092–1099.
26. Mejía RM, Linetzky B, Diez Roux A, Ferrante D, De Maio F. Socioeconomic status and tobacco consumption among adolescents: a multilevel analysis of Argentina's global youth tobacco survey. 2012.
27. Sakore DN, Parande MA, Tapare VS, Bhattacharya S. Knowledge, attitude and practice of tobacco consumption among male college students of a rural area of Pune, Maharashtra. International Journal of Community Medicine and Public Health. 2017;4: 3455.
28. Huang GC, Unger JB, Soto D, Fujimoto K, Pentz MA, Jordan-Marsh M, et al. Peer Influences: The Impact of Online and Offline Friendship Networks on Adolescent Smoking and Alcohol Use. Journal of Adolescent Health. 2014;54: 508–514. doi:10.1016/j.jadohealth.2013.07.001
29. Duko B, Melese Y, Ebrahim J. Determinants of cigarette smoking among adolescents in Ethiopia: A cross-sectional study. Tobacco induced diseases. 2019;17.
30. Ham DC, Przybeck T, Strickland JR, Luke DA, Bierut LJ, Evanoff BA. Occupation and workplace policies predict smoking behaviors: analysis of national data from the current

population survey. Journal of occupational and environmental medicine/American College of Occupational and Environmental Medicine. 2011;53: 1337.

31. Shields M. Smoking bans: influence on smoking prevalence. Health Reports. 2007;18: 9.

32. Perelman J, Alves J, Pfoertner T-K, Moor I, Federico B, Kuipers MA, et al. The association between personal income and smoking among adolescents: a study in six European cities. Addiction. 2017;112: 2248–2256.

33. Nargis N, Thompson ME, Fong GT, Driezen P, Hussain AG, Ruthbah UH, et al. Prevalence and patterns of tobacco use in Bangladesh from 2009 to 2012: evidence from International Tobacco Control (ITC) Study. PloS one. 2015;10: e0141135.

34. Lalithambigai G, Rao A, Rajesh G, Shenoy R, Pai MB. Predictors of cigarette smoking among young adults in Mangalore, India. Asian Pacific Journal of Cancer Prevention. 2016;17: 45–50.

35. Pradhan PMS, Niraula SR, Ghimire A, Singh SB, Pokharel PK. Tobacco use and associated factors among adolescent students in Dharan, Eastern Nepal: a cross-sectional questionnaire survey. BMJ open. 2013;3.

36. Abou-Faddan HH, Ahmed SM. Knowledge, Attitude and Practice Study on Smoking among Male Students in Al-Jabal Al-Gharbi University, Gharian–Libya. Journal of American Science. 2012;8: 485–491.

37. Stanton CA, Papandonatos G, Lloyd-Richardson EE, Kazura A, Shiu S-Y, Niaura R. How do mothers, fathers, and friends influence stages of adolescent smoking? Adolescent & family health: A & FH. 2009;4: 95.

38. Saari AJ, Kentala J, Mattila KJ. The smoking habit of a close friend or family member—how deep is the impact? A cross-sectional study. BMJ open. 2014;4.

39. Vink JM, Willemsen G, Engels RC, Boomsma DI. Smoking status of parents, siblings and friends: predictors of regular smoking? Findings from a longitudinal twin-family study. Twin Research and Human Genetics. 2003;6: 209–217.
40. Hassan MS, Hossain MK, Khan HT. Prevalence and predictors of tobacco smoking among university students in Sylhet Division, Bangladesh. International health. 2019;11: 306–313.
41. Kumar V, Talwar R, Roy N, Raut D, Singh S. Psychosocial determinants of tobacco use among school going adolescents in Delhi, India. Journal of addiction. 2014;2014.
42. Abughosh S, Wu IH, Hawari F, Peters RJ, Yang M, Crutchley R. Predictors of intention to quit cigarette smoking among Jordanian adults. Epidemiology. 2011;1: 1–7.
43. Dawood OT, Rashan MAA, Hassali MA, Saleem F. Knowledge and perception about health risks of cigarette smoking among Iraqi smokers. Journal of pharmacy & bioallied sciences. 2016;8: 146.
44. Semmer NK, Cleary PD, Dwyer JH, Fuchs R, Lippert P. Chapter 1: Psychosocial Predictors of Adolescent Smoking in Two German Cities: The Berlin-Bremen Study. Morbidity and mortality weekly report. 1987;36: 3S-10S.
45. Stronks K, van de Mheen HD, Looman CW, Mackenbach JP. Cultural, material, and psychosocial correlates of the socioeconomic gradient in smoking behavior among adults. Preventive medicine. 1997;26: 754–766.

Table 1: Basic characteristics of the respondents

Variables	Frequency	Percent
**Age group**		
15-17	154	40.0
18-20	111	28.8
21-24	120	31.2
**Average age (SD)**	**18.9 (3.1)**	
**Education**		
Below secondary	48	12.5
Secondary	197	51.1
Higher secondary and above	140	36.4
**Average year of schooling (SD)**	**9.6 (3.3)**	
**Education of father**		
Below secondary	169	43.8
Secondary	108	28.1
Higher secondary and above	108	28.1
**Average year of schooling of father (SD)**	**7.3 (5.4)**	
**Education of mother**		
Below secondary	207	53.8
Secondary	125	32.4
Higher secondary and above	53	13.8
**Average year of schooling of mother (SD)**	**5.9 (4.7)**	
**Occupation**		
Day labourer	20	5.2
Service	20	5.2
Business	65	16.9
Students	258	67.0
Unemployed	22	5.7
**Monthly income (in BDT)**		
No income	257	66.8
< 5000	21	5.5
≥5000	107	27.7
**Average income (SD)**	**4439 (7877.8)**	
**Family's monthly income (in BDT)**		
< 15000	97	25.2
15000-25000	177	46.0
> 25000	111	28.8
**Average monthly income of family (SD)**	**22634 (12958.1)**	
**Financially dependent on the family**		
Dependent	276	71.7
Independent	109	28.3
**Current smoking status**		
No	230	59.7
Yes	155	40.3
**Current smoking status of father***		
No	164	42.6
Yes	221	57.4
**Current smoking status of brother**		

No	317	82.3
Yes	68	17.7
**Current smoking status of close friend**		
No	246	63.9
Yes	139	36.1
**Smoking brings the risks of chronic diseases**		
No	6	1.6
Yes	379	98.4
**Smoking is harmful to other non-smokers**		
No	14	3.6
Yes	371	96.4

Notes: BDT: Bangladesh currency – Taka; SD indicates Standard Deviation; * No mother of the study participants found as smoker.

Table 2: Percentage of smokers by socio-demographics

Variables	Smoker	
	Number	Row percentage
**Age group**		
15-17	30	19.5
18-20	45	40.5
21-24	80	66.7
p-value	<0.001	
**Education**		
Below secondary	28	58.3
Secondary	53	26.9
Higher secondary and above	74	52.9
p-value	<0.001	
**Education of father**		
Below secondary	65	38.5
Secondary	42	38.9
Higher secondary and above	48	44.4
p-value	0.57	
**Education of mother**		
Below secondary	77	37.2
Secondary	54	43.2
Higher secondary and above	24	45.3
p-value	0.40	
**Occupation**		
Day labourer	16	80.0
Service	12	60.0
Business	34	52.3
Students	83	32.2
Unemployed	10	45.5
p-value	<0.001	
**Monthly income (in BDT)**		
No income	79	30.7
< 5000	13	61.9
≥5000	63	58.9
p-value	<0.001	
**Family's monthly income (in BDT)**		
< 15000	26	26.8
15000-25000	75	42.4
> 25000	54	48.6
p-value	<0.001	
**Financially dependent on the family**		
Dependent	93	33.7
Independent	62	56.9
p-value	<0.001	
**Current smoking status of father**		
No	55	33.5
Yes	100	45.2
p-value	0.02	

**Current smoking status of brother**		
No	114	36.0
Yes	41	60.3
p-value	<0.001	
**Current smoking status of close friends**		
No	54	22.0
Yes	101	72.7
p-value	<0.001	
**Smoking brings the risk of chronic disease**		
No	5	83.3
Yes	150	39.6
p-value	0.03	
**Smoking is harmful to other non-smokers**		
No	11	78.6
Yes	144	38.8
p-value	<0.001	

Notes: BDT: Bangladesh currency – Taka. The p-values are of chi-square tests.

Table 3: Association of socio-demographics with smoking status

Variables	Odds Ratio	95% confidence interval
**Age group**		
15-17®	1.00	-
18-20	1.78	0.77 – 4.11
21-24	5.54***	2.25 – 13.61
**Education**		
Below secondary®	1.00	-
Secondary	0.44	0.16 – 1.19
Higher secondary and above	0.51	0.17 – 1.53
**Occupation**		
Day labourer®	1.00	-
Service	0.40	0.06 – 2.54
Business	0.15**	0.03 – 0.69
Students	0.42	0.07 – 2.49
Unemployed	0.20	0.03 – 1.28
**Monthly income (in BDT)**		
No income®	1.00	-
< 5000	4.92***	1.87 – 12.59
≥5000	3.11	0.56 – 7.27
**Family's monthly income (in BDT)**		
< 15000®	1.00	-
15000-25000	2.09*	1.01 – 4.34
> 25000	2.77**	1.24 – 6.18
**Financially dependent on the family**		
Dependent®	1.00	-
Independent	2.62	0.59 – 7.52
**Current smoking status of father**		
No®	1.00	-
Yes	2.24***	1.25 – 3.98
**Current smoking status of brother**		
No®	1.00	-
Yes	3.03***	1.46 – 6.29
**Current smoking status of close friend**		
No®	1.00	-
Yes	10.65***	5.89 – 19.25
**Smoking brings the risk of chronic disease**		
No®	1.00	-
Yes	0.15	0.01 – 2.54
**Smoking is harmful to other non-smokers**		
No®	1.00	
Yes	0.13**	0.03 – 0.70

Notes: BDT: Bangladesh currency – Taka; ®: Reference Category; Level of significance: ***: p<0.001; **: p<0.01; *: p<0.05.
